# Physical activity interventions delivered through digital health technology for improving workers’ mental health symptoms: a systematic review and meta-analysis

**DOI:** 10.1093/joccuh/uiaf035

**Published:** 2025-06-30

**Authors:** Satoru Kanamori, Kotaro Imamura, Yuta Inagawa, Takenori Yamauchi, Hiroki Ikeda, Takuro Okuyama, Go Muto, Rika Kato, Mako Iida, Hiroki Asaoka, Akiomi Inoue, Kazuhiro Watanabe, Kanami Tsuno, Natsu Sasaki, Yuka Kobayashi, Asuka Sakuraya, Yu Komase, Yasumasa Otsuka, Mai Iwanaga, Reiko Inoue, Kazuto Kuribayashi, Ayako Hino, Akihito Shimazu, Akizumi Tsutsumi, Norito Kawakami, Naomichi Tani, Hisashi Eguchi, Noriko Kojimahara, Takeshi Ebara

**Affiliations:** Graduate School of Public Health, Teikyo University, Itabashi, Japan; Department of Preventive Medicine and Public Health, Tokyo Medical University, Shinjuku-ku, Japan; Department of Digital Mental Health, Graduate School of Medicine, The University of Tokyo, Bunkyo-ku, Japan; Department of Psychiatry, Jichi Medical University, Shimotsuke, Japan; Department of Hygiene, Public Health and Preventive Medicine Showa Medical University, School of Medicine, Shinagawa-ku, Japan; National Institute of Occupational Safety and Health, Japan Organization of Occupational Health and Safety, Kawasaki, Japan; Graduate School of Public Health, Teikyo University, Itabashi, Japan; Department of Hygiene, Kitasato University School of Medicine, Sagamihara, Japan; Department of Psychiatry, Jichi Medical University, Shimotsuke, Japan; Department of Mental Health, Graduate School of Medicine, The University of Tokyo, Bunkyo-ku, Japan; Department of Mental Health, Graduate School of Medicine, The University of Tokyo, Bunkyo-ku, Japan; Institutional Research Center, University of Occupational and Environmental Health, Japan, Kitakyushu, Japan; Department of Public Health, Kitasato University School of Medicine, Sagamihara, Japan; School of Health Innovation, Kanagawa University of Human Services, Yokosuka, Japan; Department of Mental Health, Graduate School of Medicine, The University of Tokyo, Bunkyo-ku, Japan; Department of Clinical Psychology, Faculty of Social Policy & Administration, Hosei University, Chiyoda, Japan; Department of Digital Mental Health, Graduate School of Medicine, The University of Tokyo, Bunkyo-ku, Japan; Healthcare Business Division, Fujitsu Japan limited, Kawasaki, Japan; Institute of Human Sciences, University of Tsukuba, Tsukuba, Japan; Department of Community Mental Health & Law, National Institute of Mental Health, National Center of Neurology and Psychiatry, Kodaira, Japan; Department of Public Health, Kitasato University School of Medicine, Sagamihara, Japan; Department of Psychiatric Nursing, Division of Nursing, Chiba Faculty of Nursing, Tokyo Healthcare University, Shinagawa-ku, Japan; Department of Mental Health, Institute of Industrial Ecological Sciences, University of Occupational and Environmental Health, Japan, Kitakyushu, Japan; Faculty of Policy Management, Keio University, Fujisawa, Japan; Department of Public Health, Kitasato University School of Medicine, Sagamihara, Japan; Department of Digital Mental Health, Graduate School of Medicine, The University of Tokyo, Bunkyo-ku, Japan; Department of Ergonomics, Institute of Industrial Ecological Sciences, University of Occupational and Environmental Health, Japan, Kitakyushu, Japan; Department of Mental Health, Institute of Industrial Ecological Sciences, University of Occupational and Environmental Health, Japan, Kitakyushu, Japan; Section of Epidemiology, Shizuoka Graduate University of Public Health, Shizuoka, Japan; Department of Ergonomics, Institute of Industrial Ecological Sciences, University of Occupational and Environmental Health, Japan, Kitakyushu, Japan

**Keywords:** Ehealth, exercise, depression, perceived stress, attrition, adherence

## Abstract

**Objectives:**

This study aimed to clarify the primary preventive effects of physical activity interventions delivered through digital health technology (DHT) on workers’ mental health symptoms, and to examine the conditions of attrition and adherence in these interventions.

**Methods:**

We examined randomized controlled trials (RCTs) that analyzed the effects of physical activity interventions delivered through DHT on workers’ health outcomes. We included RCTs published in English or Japanese since 2010 and excluded studies that targeted specific diseases or secondary and tertiary prevention. We conducted the search on July 25, 2023, using Cochrane CENTRAL, Embase, PsycINFO, PubMed, and Ichushi-Web, and citation searches. We assessed risk of bias using the Cochrane Risk of Bias Tool version 2, and data were integrated using a random-effects model. Attrition rates were averaged, and adherence was qualitatively reviewed.

**Results:**

Eight studies were included in the systematic review, and 5 in the meta-analysis. Pooled effect sizes immediately after intervention were as follows: Cohen *d* = −0.51 (95% CI, −0.75 to −0.27) for depression and negative affect, and −0.36 (95% CI, −0.60 to −0.05) for perceived stress. The attrition rate was 16.8% and 12.4% for the control and intervention groups, with only 2 studies providing details on adherence.

**Conclusions:**

Physical activity interventions delivered through DHT may moderately improve depression and negative affect, and slightly reduce perceived stress among workers. However, these findings should be interpreted with caution due to the limited number of studies and low evidence certainty. Future studies should explore long-term effects, additional mental health outcomes, and factors affecting adherence.

## Teaser

Key points

**What is already known on this topic:** Digital health technology (DHT)-based interventions are rapidly advancing as a primary prevention strategy for mental health. However, the primary preventive effects of physical activity interventions delivered through DHT on mental health symptoms among workers, as well as the actual conditions of attrition and adherence, remain unclear.

**What this study adds:** This study found moderate effects on depression and negative affect immediately after the interventions, and small to moderate effects on perceived stress. The attrition rate immediately after the intervention was approximately 1 in 6 for the intervention group, with limited descriptions of adherence.

**How this study might affect research, practice, or policy:** Although some degree of attrition is expected in primary prevention of depression, negative affect, and stress among workers, implementing physical activity interventions through DHT may be effective.

## 1. Introduction

In recent years, mental health issues among workers have gained significant attention as a critical challenge in global occupational health. It is estimated that 15% of working-age adults experience a mental disorder at some point in their lives.^1^ Symptoms such as anxiety and depression are among the most common mental health conditions. In 2019, an estimated 310 million people worldwide lived with anxiety, and 280 million suffered from depression,^1^ highlighting the need for effective interventions as primary prevention measures.

Regular physical activity has been increasingly recognized as a critical primary preventive measure for reducing the risk of mental disorders among workers. Regular physical activity has been shown to reduce depression, anxiety, and psychological distress,^2^ and is effective in the primary prevention of general mental health disorders.^3^ Consequently, regular physical activity is recommended as an accessible and effective approach for maintaining workers’ psychological well-being and is included in preventive strategies to promote occupational health.

In recent years, interventions leveraging digital health technology (DHT) to promote physical activity among workers have gained significant support, reflecting a growing trend in workplace health promotion strategies. DHT has the potential to address several challenges in health care systems, including poor geographical accessibility, low demand for services, and financial costs.^4^ The effectiveness of DHT interventions aimed at increasing physical activity has been demonstrated through umbrella reviews and meta-analyses.^5^ DHT tools that promote physical activity include pedometers, accelerometers, text messages, and websites.^6^ These tools provide behavioral change techniques, including personalized goal setting, real-time feedback, gamification elements, and peer support functions.^7^ Furthermore, several studies have examined the effects of DHT-based physical activity interventions on mental health symptoms among workers. Examples include an online sedentary behavior modification program^8^ and mobile phone applications (apps) combined with email.^9^ However, to our knowledge, no systematic review has integrated these findings.

Additionally, DHT-based interventions have been criticized for their high attrition rates and low adherence. A systematic review of randomized controlled trials (RCTs) of occupational mental health interventions reported attrition rates of 14.5% in the intervention groups at post-intervention assessment for face-to-face interventions, compared with 31.7% for online interventions,^10^ highlighting the notably higher attrition rates associated with online interventions. Similarly, the attrition rate for smartphone-delivered interventions for mental health problems was 24.1% at short-term follow-up and 35.5% at long-term follow-up.^11^ These rates were slightly higher compared with the results from meta-analyses of face-to-face therapies, which reported attrition rates of 19.9% for individual psychotherapy for depression^12^ and 17.0% for anxiety disorders.^13^ Regarding adherence in a key DHT intervention using a mental health app, the median app opening rates were 69.4% on day 1, 3.9% on day 15, and 3.3% on day 30, relative to users who opened the app on day 0.^14^ Likewise, a systematic review of mobile health interventions aimed at promoting physical activity and reducing sedentary behavior in the workplace showed that, although initial acceptance was high, technology use and engagement decreased significantly over time.^7^ These findings suggest that, although high attrition rates and low adherence are expected, the specific conditions of attrition and adherence in DHT-based physical activity interventions for the primary prevention of mental health symptoms among workers remain unclear.

The purpose of this study was to examine the primary preventive effects of DHT-based physical activity interventions on mental health symptoms among workers. Additionally, the study aimed to clarify the actual conditions of attrition and adherence in such interventions.

## 2. Materials and methods

This study was conducted as part of the Developing Minds-compliant Guidelines for General Preventive Intervention Using Digital Health Technologies for Mental Health (DeLiGHT) project.^15^ The systematic review and meta-analysis were carried out following the Preferred Reporting Items for Systematic Reviews and Meta-Analyses (PRISMA) guidelines.^16^ The study was registered with the University Hospital Medical Information Network (UMIN) Clinical Trials Registry (UMIN000051630, UMIN000051631). Of the 2 registrations, the first concerned attrition and adherence, whereas the second focused on the effectiveness of improving mental health outcomes.

### 2.1. Eligibility criteria

In accordance with the DeLiGHT project, a comprehensive systematic review was conducted to examine the effects of DHT-based interventions on the primary prevention of mental health issues among workers. This study, as a part of the review, specifically focused on how physical activity interventions affect mental health symptoms.

In the comprehensive systematic review, the population (P) consisted of workers, the interventions (I) included nonpharmacological approaches using DHT (eg, physical activity, nutrition, lifestyle counseling, and cognitive behavioral interventions), the comparison group (C) involved any control condition without specific restrictions, and the outcomes (O) focused on mental health, positive mental health, and work-related outcomes.

The following studies were included: (1) those targeting workers, (2) those implementing a DHT intervention, (3) those evaluating health outcomes, (4) those employing an RCT design, and (5) those published after 2010. Since 2010, the proliferation of smartphones and wearable devices has led to significant growth and development in DHT research. Therefore, limiting the search to studies published from 2010 onward was likely to yield the most up-to-date evidence reflecting current technological trends and usage patterns. Although including studies published before 2010 might have increased the number of eligible articles, we focused on studies from 2010 onward to ensure the relevance of the findings and avoid outdated technologies no longer in use. This approach is also supported by recent literature^17^ and guidelines,^4^ which highlight the rapid evolution of mobile- and AI-based digital health technologies over the past decade. This global trend has also been reflected in national information and communication technology usage patterns, such as in Japan, where internet access via mobile devices surpassed access via personal computers around 2010.^18^

The following exclusion criteria were applied: (1) studies targeting workers with specific diseases, (2) studies including participants who were not workers, (3) studies using interventions aimed at secondary or tertiary prevention, (4) studies evaluating treatment effects for specific diseases or symptoms, (5) studies that were not original research papers, and (6) studies published in languages other than English or Japanese.

In this meta-analysis, the population (P) consisted of workers, the intervention (I) involved physical activity programs incorporating DHT, the comparison group (C) included any control condition without specific restrictions, and the primary outcomes (O) targeted a range of mental health symptoms (eg, depression, anxiety, negative affect, perceived stress, traumatic stress reactions, and sleep quality). Notably, P and C were identical to those defined in the comprehensive systematic review, whereas I and O addressed concepts that were included as part of the definitions used in the review. Therefore, based on the literature extracted from the comprehensive systematic review, we selected studies that matched the PICO of this study.

The definition of DHT services in this study is as follows^19^:

“DHT service is defined as ‘healthcare services aimed at primary prevention provided to the general workforce using information and communication technology (ICT) and digital technology.’ DHT includes services that use technical algorithms (eg, apps, communication robots, wearable devices, information provision through noncontact sensing devices, self-monitoring, real-time feedback), as well as services that do not use technical algorithms (eg, online counseling that relies solely on internet-based means). The targets of digital mental health services are to prevent mental health issues, improve quality of life (QOL) and functionality (productivity, absenteeism, etc), enhance positive mental health (work engagement, well-being, etc), prevent suicide, and prevent substance abuse such as alcohol and drugs, by using DHT. The DHT service for mental health does not cover medical purposes.”

### 2.2. Information sources and search strategy

The databases used for the search were Cochrane CENTRAL, Embase, PsycINFO/ARTICLES, PubMed, and Ichushi-Web. The search date was July 25, 2023. Additionally, citation searches were conducted based on the studies that were included.

The search strategy for the comprehensive systematic review used keywords corresponding to the “P,” “I,” and “O.” The search terms for “I” were selected based on a prior umbrella review on DHT,^17^ and those for “O” were chosen based on existing guidelines on mental health at work.^1^ The search strategy is described in detail in Appendix 1.

### 2.3. Selection process

In this comprehensive systematic review conducted in accordance with the DeLiGHT project, the following steps were undertaken during the initial screening of articles extracted from each database:

1. Eligibility criteria (PICO of the included studies and exclusion criteria) were assessed based on the title and abstract of the article.

2. If eligibility could not be determined from the abstract, the study was provisionally included.

For the secondary screening, we reviewed the full texts to determine whether the studies met the inclusion criteria.

In this study, physical activity interventions were defined as interventions aimed at increasing physical activity or reducing sedentary behavior, based on the World Health Organization (WHO) guidelines on physical activity and sedentary behavior.^20^ Interventions primarily focusing on approaches other than promoting physical activity or reducing sedentary behavior or those incorporating these elements only as minor components were excluded. There has been inconsistency in previous studies regarding whether yoga should be classified as a form of physical activity.^21^ However, yoga is recommended as a form of physical activity in the WHO physical activity guidelines.^20^ Therefore, this study included yoga as a physical activity intervention. Based on these definitions, studies involving physical activity interventions were selected from those included in the secondary screening.

During the primary and secondary screenings, 2 independent reviewers selected the studies. If discrepancies occurred between their decisions during the primary screening, the studies were provisionally included. Disagreements arising during the secondary screening were resolved by the researchers through discussion. Subsequently, from the articles identified in the comprehensive systematic review, studies involving physical activity interventions were selected based on the intervention classifications determined during the secondary screening.

### 2.4. Data collection process and data items

The extracted items included the title, author names, publication year, study design, PICO, intervention duration, follow-up timing, attrition, adherence, and outcome-related information. Referring to previous studies on attrition and adherence in smartphone-based mental health interventions,^11^ the attrition rate was defined as the proportion of randomized participants who did not complete the follow-up evaluations. We also calculated a weighted average for the attrition rate, considering the number of participants in both the intervention and control groups. Adherence refers to the extent to which participants use or engage with the intervention—for example, the number of logins, time spent on the intervention, or the proportion of participants who completed all activities/modules.^11^

After the secondary screening, data collection for the studies confirmed for inclusion was conducted by 2 independent reviewers. Discrepancies were resolved through discussion between the reviewers until consensus was reached. No automation tools were employed in this process, and the researchers managed the data using Excel.

### 2.5. Study risk-of-bias assessment

For the risk-of-bias assessment in the meta-analysis, we used version 2 of the Cochrane risk-of-bias tool for randomized trials (RoB 2).^22^ This tool analyzes 5 domains of bias: bias from the randomization process, bias due to deviations from intended interventions, bias from missing outcome data, bias in outcome measurement, and bias in the selection of the reported result. Each domain includes a set of signaling questions with responses that determine the risk of bias (low risk, some concerns, and high risk). Two independent reviewers conducted the evaluations. When their judgments differed, the final decision was reached through discussions among the researchers.

**Figure 1:**
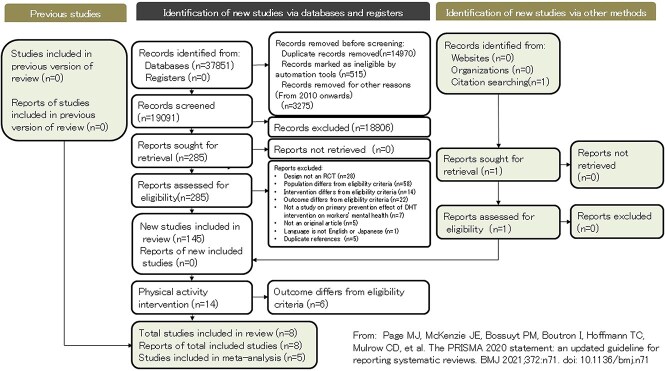
Flow diagram.

### 2.6. Effect measures and synthesis methods

Based on the mean and SD at baseline and follow-up for both the intervention and control groups, or the mean change and its SD or CI, we calculated Cohen *d* and its 95% CI as the effect size for each outcome category. We evaluated the effect size using Cohen *d*, considering 0.2 as a small effect, 0.5 as a medium effect, and 0.8 as a large effect. We excluded studies that did not provide the necessary data for integration from the meta-analysis.

This review comprehensively examined mental health symptoms as outcomes; however, the number of outcomes available for meta-analysis was limited. To address this limitation, the outcomes measured in each study were screened and grouped based on their conceptual similarities. As a result, the outcomes were classified into 2 primary categories: measures that primarily assessed “depression and negative affect” and those that primarily assessed “perceived stress.” The classification process entailed 3 main steps. First, priority was given to measures reported to have validity and reliability. Next, in studies reporting multiple outcomes, those that could be integrated with outcomes from other studies or that employed similar measurement scales were selected. Finally, for outcomes representing the same concept, scales commonly used across multiple studies were prioritized. Following this classification, a meta-analysis was conducted for each category to calculate an integrated effect size estimate.

A meta-analysis was conducted only when at least 3 studies evaluated the same outcome. Given the expected heterogeneity due to differences in regions or populations among the included studies, we used a random-effects model for data integration.^23^ To assess heterogeneity, we generated forest plots to visually examine the variation in effect sizes across studies. Additionally, the *I*^2^ statistic and the *P* value for heterogeneity were calculated to quantify the degree of heterogeneity. The *I*^2^ statistic indicates low, moderate, and high heterogeneity at *I*^2^ values of 25%, 50%, and 75%, respectively.^24^ To explore the causes of heterogeneity and assess the robustness of the integrated results, we performed a leave-one-out sensitivity analysis. Statistical analyses were conducted using SPSS Statistics (version 29), setting the 2-tailed significance level at 5%.

### 2.7 Reporting bias assessment and certainty assessment

To assess the presence of reporting bias, we created funnel plots to visually inspect for asymmetry^25^ and conducted the Egger test to statistically examine this bias.^26^ We used the Grading of Recommendations Assessment, Development, and Evaluation (GRADE) approach to evaluate the certainty of the evidence.^27^ For each outcome, we assessed the risk of bias, inconsistency, indirectness, imprecision, and reporting bias, and rated the overall quality of the evidence as “high,” “moderate,” “low,” or “very low.”

## 3. Results

### 3.1. Selection and characteristics of the studies

Figure 1 presents a flowchart depicting the number of records identified in the search, the number of studies included in the review, and the number of studies included in the meta-analysis. The total number of records identified from each database was 11 680 from Cochrane CENTRAL, 12 780 from Embase, 2899 from PsycINFO/ARTICLES, 9221 from PubMed, and 1271 from Ichushi-Web, giving a total of 37 851 records. Of these, 14 970 duplicate records, 515 records marked as ineligible by the automation tool, and 3275 reports published before 2009 were excluded, leaving 19 091 records. A primary screening of these remaining records resulted in 285 studies being included for further analysis.

The following studies were excluded during the secondary screening: 28 studies lacking an RCT design, 58 including participants who did not meet the eligibility criteria, 14 including interventions that did not meet the criteria, 22 studies with outcomes that did not meet the criteria, 7 investigating the primary prevention effects of physical activity interventions delivered through DHT on workers’ mental health, 5 studies that were not original articles, and 1 that was published in a language other than English or Japanese. Additionally, we removed 5 duplicate references. After the exclusions, 145 studies remained and were included in the comprehensive systematic review following the DeLiGHT project guidelines.

Of these, 14 studies focused on physical activity interventions, and 8 studies^8^^,^^9^^,^^28^^-^^33^ matched the PICO criteria for this research. However, 2 studies^9^^,^^30^ were excluded from the meta-analysis due to the lack of data necessary for their inclusion, and one study^31^ was excluded because its outcome (sleep quality) was not reported in 3 or more studies, which is the minimum required for meta-analysis. Consequently, 5 studies^8^^,^^28^^,^^29^^,^^32^^,^^33^ were included in the meta-analysis.

Table 1 presents the characteristics of the studies included. One study was published in 2011,^32^ 1 in 2015,^30^ 2 in 2020,^29^^,^^33^ 1 in 2021,^28^ 1 in 2022,^8^ and 2 in 2023.^9^^,^^31^ The study designs comprised five RCTs,^28^^-^^32^ one 3-arm RCT,^9^ one 4-arm RCT,^8^ and one cluster RCT.^33^ Regarding geographic distribution, 3 studies were conducted in the United States,^8^^,^^32^^,^^33^ 3 in the United Kingdom,^28^^-^^30^ 1 in Spain,^9^ and 1 in Turkey.^31^ Sample sizes varied from a minimum of 18 participants^30^ to a maximum of 553 participants.^33^

**Table 1:** Characteristics of the studies.

**Study**	**Design**	**Meta-analysis**	**Participants**	**Intervention**	**Control**	**Outcome (only mental health symptoms and disorders)**	**Intervention period**	**Follow-up (attrition rate** ^**a**^ ^**s**^ **)**	**Type of assessment of physical activity outcomes and findings**	**Results**
Falk et al (2022)^8^	RCT (4-arm)	Included	*n* = 95 Sedentary university employees working from home in the United States	“Varidesk”: height-adjustable desk provision + online sedentary behavior modification program (Primary DHT: online behavioral modules)	Waiting list	Negative affect (Positive and Negative Affect Schedule: PANAS) and stress (Perceived Stress Scale: PSS)	12 weeks	At the end of the intervention period (8.7%; 2/23)	Qualitative assessment; increased desk-time physical activity and off-duty exercise	No significant improvement in negative affect and stress
Wadhen and Cartwright (2021)^28^	RCT	Included	*n* = 34 Employees working from home in the United Kingdom	The online streamed yoga (Primary DHT: Zoom online meeting)	Waiting list	Depression, anxiety, stress (Depression, Anxiety & Stress scale; DASS-21), and stress (Perceived Stress Scale-14 items; PSS-14)	6 weeks	At the end of the intervention period (34.6%; 9/26)	Qualitative assessment; physical activity level not assessed	Significant improvements in depression and perceived stress, but not stress and anxiety
Carter et al (2020)^29^	RCT	Included	*n* = 18 Office-based workers from one university in the United Kingdom	“Exertime” is designed to prompt employees to interrupt prolonged sitting with brief bouts of PA during work hours and is based on the habit formation theory (Primary DHT: the e-health computer-based software and email)	Waiting list	Negative affect (Positive and Negative Affect Schedule; PANAS) and stress (Health and Work Questionnaire; HWQ)	8 weeks	At the end of the intervention period (0.0%; 0/6)	Quantitative assessment; large reductions in sitting time and increases in standing and stepping during work hours	Between-group differences were 0.4 ± 12.0 for negative affect (Cohen *d* = 0.05) and 0.3 ± 3.9 for stress (Cohen *d* = 0.04)
Thøgersen-Ntoumani et al (2015)^30^	RCT	Not	*n* = 56 Physically inactive employees from a large university in the United Kingdom	Group-led lunchtime walks and independently organized walks (Primary DHT: the mobile phone program)	Waiting list	Nervousness (Job Affect Scale)	10 weeks	At the end of the intervention period (25.7%; 9/35)	Qualitative assessment; physical activity level not assessed	No significant improvement in nervousness

(Continued)

**Table 1:** Continued

**Study**	**Design**	**Meta-analysis**	**Participants**	**Intervention**	**Control**	**Outcome (only mental health symptoms and disorders)**	**Intervention period**	**Follow-up (attrition rate** ^**a**^ ^**s**^ **)**	**Type of assessment of physical activity outcomes and findings**	**Results**
Díaz-Silveira et al (2023)^9^	RCT (3-arm)	Not	*n* = 94 Employees at the headquarters of 2 large multinational corporations in the service sector in Spain	Aerobic physical exercise at a moderate to high intensity (Primary DHT: a mobile app and email)	Waiting list	Daily stress symptoms (a single item) and daily sleep quality (a single item from the Pittsburgh Sleep Quality Index)	22 working days	Post-test (details unknown) (10.0%; 3/30)	Qualitative assessment; physical activity level not assessed	Significant improvements in daily stress symptoms
Sis Çelik and Yarali (2023)^31^	RCT	Not	*n* = 100 Nurses working in a hospital in Turkey	Laughter yoga sessions by group (Primary DHT: Zoom and WhatsApp)	Waiting list	Sleep quality (Pittsburgh Sleep Quality Index)	4 weeks	At the end of the intervention period (8.0%; 4/50)	Qualitative assessment; physical activity level not assessed	Significant improvements in sleep quality
Irvine et al (2011)^32^	RCT	Included	*n* = 221 Employees at a large manufacturing plant in the United States	Get Moving: a repeat-visit website providing information and support to develop a personalized physical activity plan (Primary DHT: website)	No intervention	Depression (2 items from the SF-12 Mental Health subscale, a single item from the SF-36 Mental Health scale, and a single item), anxiety (a single item from the SF-36 Mental Health scale), and stress (a single item)	28 days	At the end of the intervention period (13.5%; 15/111)	Quantitative assessment; significant increase in physical activity duration and stage of change	Significant improvements in depression, anxiety, and stress
Linnan et al (2020)^33^	Cluster RCT	Included	*n* = 553 Childcare workers from 56 childcare centers in the United States	“Healthy Lifestyles”: a 6-month, multi-level, theory-guided intervention designed to increase physical activity and improve other health behaviors among childcare workers (Primary DHT: electronic messaging and an interactive website)	“Healthy Finances”: a program designed to provide a similar level of attention as the Healthy Lifestyles arm. The critical difference was that all messages focused on workers’ financial well-being and the center’s financial success	Distress (a single item) and sleep quality (a single item)	6 months	At the end of the intervention period (18.8%; 47/250) and 18 months (no data)	Quantitative assessment; no significant improvement in physical activity	No significant improvement in distress

Abbreviations: DHT, digital health technology; RCT, randomized controlled trial.

a Attrition rate for the main intervention group for physical activity.

Three intervention studies focused on the promotion of physical activity:

1. A 15 to 30-minute aerobic exercise program during lunch breaks aiming to reduce stress symptoms and fatigue while improving sleep quality.^9^ As part of the DHT intervention, a mobile application was used to record activity status, and email was used for communication with the instructor.
2. The “Get Moving” website provided personalized physical activity plans for sedentary employees, aiming to enhance motivation and knowledge.^32^
3. The CARE study implemented a multi-level, workplace-based intervention targeting childcare staff, with the goal of promoting physical activity.^33^ The DHT intervention included an interactive website for goal setting and self-monitoring, as well as email communication for providing customized feedback.

Two intervention studies focused on the promotion of yoga:

1. A 6-week online yoga program aiming to reduce stress and enhance the well-being of individuals working from home.^28^ The intervention used DHT, such as live-streamed sessions and online platforms for participation, engagement, and instructor interaction.
2. A laughter yoga program was conducted to improve the psychological resilience and sleep quality of nurses.^31^ Zoom was used to conduct the laughter yoga sessions, whereas WhatsApp facilitated fast and easy communication among the experimental group, including session-related discussions.

One intervention study focused on promoting walking:

1. A 10-week program implemented 30-minute group-led lunchtime walks 3 times a week to enhance workplace relaxation and enthusiasm among physically inactive employees.^30^ DHT was used to support the intervention through a poll for walk sign-ups, motivational text messages to enhance engagement, and programmed mobile phones that randomly scheduled measurement days and sent alarms for assessments of job affect and workload.

Two intervention studies focused on the reduction of sedentary behavior:

1. A height-adjustable desk and an online behavioral intervention program were implemented in a remote work setting to reduce sedentary time and enhance mental health and productivity.^8^
2. An e-health intervention for office workers used computer-based prompts to interrupt prolonged sitting, aiming to increase physical activity and improve vascular function.^29^

The types of DHT used in the interventions were electronic messaging (5 studies),^8^^,^^9^^,^^30^^,^^31^^,^^33^ websites (2 studies),^32^^,^^33^ online meetings or counseling (2 studies),^28^^,^^31^ apps (2 studies),^9^^,^^31^ and computer-based software (1 study).^29^ The control groups were either waiting lists or had no intervention (7 studies),^8^^,^^9^^,^^28^^-^^32^ and active controls (1 study).^33^ The outcomes examined included perceived stress (6 studies),^8^^,^^9^^,^^28^^,^^29^^,^^32^^,^^33^ depression (2 studies),^28^^,^^32^ anxiety (2 studies),^28^^,^^32^ negative affect (2 studies),^8^^,^^29^ sleep quality (3 studies),^9^^,^^31^^,^^33^ and nervousness (1 study).^30^ The durations of the interventions ranged from 28 days^32^ to 6 months.^33^ Follow-up assessments took place immediately after the interventions in all 8 studies,^8^^,^^9^^,^^28^^-^^33^ with 1 study also conducting a follow-up 18 months later.^33^ All 8 studies^8^^,^^9^^,^^28^^-^^33^ reported attrition rates immediately following the intervention. In studies with 3 arms^9^ or 4 arms,^8^ the primary group for the DHT-based exercise intervention was designated as the intervention group (Table 1). The attrition rates immediately post-intervention ranged from 0.0% to 34.6% for the intervention groups and from 1.7% to 34.6% for the control groups. The weighted average attrition rates were 16.8% for the intervention group and 12.4% for the control group. Two studies^9^^,^^28^ reported on adherence: in a yoga intervention, all participants attended the minimum recommended number of sessions, with an average attendance of 16.6 sessions.^28^ In an aerobic physical exercise intervention, 9 of 30 participants in the intervention group did not complete 70% of the sessions.^9^ Among the 3 studies^29^^,^^32^^,^^33^ that quantitatively measured physical activity outcomes, 2 studies^29^^,^^32^ reported significant improvement in physical activity levels or reduction of sedentary time. The other studies provided only qualitative findings.

### 3.2. Risk of bias

**Figure 2:**
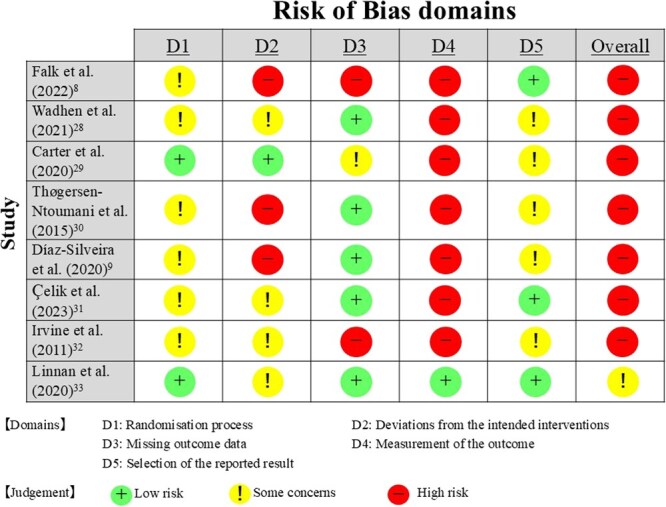
Risk of bias.

Figure 2 shows the risk of bias for the 8 studies^8^^,^^9^^,^^28^^-^^33^ included in the systematic review. None of the studies achieved a “low risk” rating for any of the 5 aspects of bias assessment. Specifically, in measuring the outcome, 7 studies^8^^,^^9^^,^^28^^-^^32^ of the 8 included studies received a “high risk” rating. Overall, 7 studies^8^^,^^9^^,^^28^^-^^32^ were categorized as “high risk,” and 1 study^33^ raised “some concerns.”

### 3.3 Results of integration

A meta-analysis was conducted using data from 5 studies that examined depression and negative affect, and perceived stress. Two of these studies investigated both depression and anxiety as outcomes,^28^^,^^32^ but neither specified the priority of these outcomes. In 1 of these studies,^32^ anxiety was assessed using a single-item measure from the Medical Outcomes Study Short-Form 36-Item Health Survey (SF-36) related to feeling “calm and peaceful.” This single item may not adequately capture the multifaceted nature of anxiety and may present significant limitations in its measurement validity. Conversely, the 3-item measure for depression had undergone validation and was deemed more robust and reliable for assessing this outcome. This methodological consideration further justified the focus on depression over anxiety. Consequently, the other study was also analyzed based on its depression results.^28^ Negative affect was measured using the Positive and Negative Affect Schedule (PANAS) in 2 studies.^8^^,^^29^ Depression and negative affect are considered closely related concepts that share a common core of general distress.^34^ Due to this shared core, we integrated both constructs in our meta-analysis.

For perceived stress, 1 study employed both the Perceived Stress Scale and subscales from the Depression, Anxiety & Stress Scale.^28^ The data from the Perceived Stress Scale, also used in another study,^8^ were selected for inclusion in the meta-analysis.

**Figure 3:**
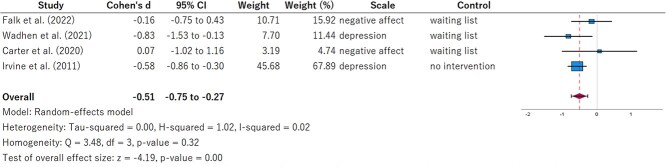
Forest plot of depression and negative affect.

**Figure 4:**
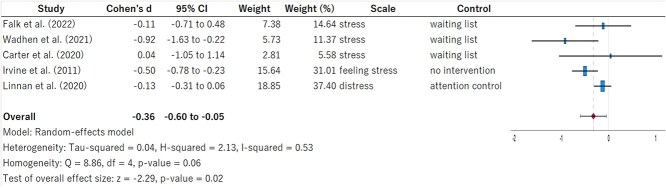
Forest plot of perceived stress.

The results of the random-effects model for depression and negative affect are shown in Figure 3. The pooled effect size (Cohen *d*) was −0.51 (95% CI, −0.75 to −0.27), indicating a significant decrease in the intervention group receiving physical activity interventions through DHT (*P* < .01). Heterogeneity was low (*I*^2^ = 2%, *P* = .32). The results of the random-effects model for perceived stress are shown in Figure 4. Cohen *d* was −0.36 (95% CI, −0.60 to −0.05), showing a marginal decrease in the intervention group (*P* = .02). Heterogeneity was moderate (*I*^2^ = 53%, *P* = .06). Appendices 2 and 3 show the results of the sensitivity analysis using leave-one-out meta-analysis. For depression and negative affect, the significant difference disappeared (*P* = .17) only when the study by Irvine et al^32^ was excluded. For perceived stress, the significant difference disappeared when either the study by Wadhen and Cartwright^28^ or Irvine et al^32^ was excluded.

### 3.4. Reporting bias

The funnel plots for each meta-analysis are shown in Appendix 4. The result of the Egger test for depression and negative affect was not significant (*P* = .18). Similarly, no significant difference was observed for perceived stress (*P* = .40).

### 3.5. Overall certainty of evidence

Overall certainty of the evidence was assessed using the GRADE approach. For depression and negative affect, all the studies analyzed were RCTs, which initially gave the evidence a “high” certainty level. However, the risk of bias was rated as “very serious” due to numerous methodological limitations, including the lack of objective measurements and absence of placebo controls in the comparison groups. In contrast, inconsistency, imprecision, indirectness, and reporting bias were assessed as presenting “no serious concerns.” As a result, the overall certainty of the evidence for depression and negative affect was classified as “low.”

For perceived stress, the same set of RCTs initially justified a “high” level of certainty. However, the risk of bias was again rated as “very serious,” due to similar methodological limitations. Inconsistency was rated as “serious” due to “moderate” heterogeneity. Indirectness, imprecision, and reporting bias presented “no serious concerns.” Consequently, the overall certainty of the evidence for perceived stress was ultimately rated as “very low.”

## 4. Discussion

This study aimed to examine the effects of primary prevention on workers’ mental health symptoms through physical activity interventions delivered through DHT. The study selection process yielded 8 studies for the systematic review, and 5 for the meta-analysis. The meta-analysis revealed that the pooled effect size for depression and negative affect immediately after the intervention was moderate, whereas the effect size for perceived stress ranged from small to moderate. The overall certainty of the evidence was assessed as “low” for depression and negative affect, and “very low” for perceived stress. Additionally, the weighted averages of the attrition rate immediately after the intervention for the 8 studies were 16.8% for the intervention group and 12.4% for the control group. Regarding adherence, only 2 studies^9^^,^^28^ reported related information, including the percentage of participants attending recommended sessions and the average number of sessions attended.

The results of the meta-analysis suggest that physical activity interventions delivered through DHT for workers may produce a moderate effect size in reducing depression and negative affect. Previous meta-analyses have shown that physical activity can decrease depression, anxiety, and psychological distress,^2^ supporting the findings of this study. Additionally, this study offers new insights by demonstrating that DHT-based physical activity interventions yield similar outcomes. These interventions were found to increase physical activity,^5^ which may have led to improvements in depression and negative affect. In the sensitivity analysis, the significance of these results disappeared only when the study by Irvine et al^32^ was excluded. Compared with the other 3 studies,^8^^,^^28^^,^^29^ this study may have produced these results due to its unique characteristic of having the largest number of participants. Notably, all 4 studies analyzed had a high risk of bias, making it difficult to evaluate the impact of excluding their results. Moreover, the overall certainty of the evidence was rated as low, and the robustness of the meta-analytic findings was limited. Therefore, these results should be interpreted with caution, particularly given the small number of included studies and the influence of individual studies on the pooled estimates. Future research should prioritize the accumulation of higher-quality studies.

The results of the meta-analysis on perceived stress revealed a small to medium effect size. Compared with a previous overview of systematic reviews,^2^ which reported that physical activity interventions for adults had a medium effect on psychological distress (median effect size = −0.60), the direction of association in this study was consistent, though the effect size was somewhat weaker. This discrepancy may be attributable to the present study’s focus on primary prevention targeting workers and interventions using DHT. Sensitivity analyses indicated that the exclusion of either Wadhen and Cartwright^28^ or Irvine et al^32^ led to a loss of statistical significance, suggesting that these studies had a considerable impact on the overall results. The physical activity intervention in Wadhen and Cartwright^28^ involved online streamed yoga, which may have been particularly effective compared with other forms of physical activity. Yoga has been identified as a potentially effective physical activity intervention for stress reduction in systematic reviews of health care workers.^35^ In addition to physical activity, yoga incorporates breath work and meditation, which are believed to act on both psychological and biological pathways, making it particularly effective for stress reduction.^36^ Furthermore, the unique circumstances of working from home during the COVID-19 outbreak might have amplified its stress-reducing effects. In contrast, the physical activity intervention in Irvine et al^32^ involved a repeat-visit website providing information and support for developing a personalized physical activity plan. The large effect size observed in this study statistically underscores the potential effectiveness of certain technological interventions in reducing stress. These 2 studies included factors distinct from other studies, suggesting that the effectiveness of the interventions may be pronounced only under specific conditions. Moreover, the overall certainty of the evidence for perceived stress was rated as very low. Given the small number of included studies and the substantial influence of individual studies, these findings should be interpreted with caution.

The overall certainty of the evidence was rated as “low” for depression and negative affect, and “very low” for perceived stress. The risk of bias was assessed as “very serious” for both outcomes. In this category, the “outcome measurement” was evaluated as “high risk” in 4^8^^,^^28^^,^^29^^,^^32^ of the 5 studies. Due to the nature of physical activity interventions delivered through DHT, maintaining blinding of control groups regarding intervention allocation is challenging. Moreover, when analyzing mental health outcomes, objective measures (eg, biomarkers^37^) are limited. For these reasons, the overall certainty of evidence is often rated as low. Furthermore, for perceived stress, inconsistency was rated as “serious” due to moderate heterogeneity. In comparison with the literature on depression and negative affect, only the study by Linnan et al^33^ differed significantly. Contributing factors to this difference may include the unique characteristics of the participants (childcare workers), the use of an alternative intervention (“Healthy Finances”) in the control group, and the extended intervention period of 6 months. Given these limitations, the overall certainty of the evidence should be interpreted with caution. However, physical activity interventions delivered through DHTs may offer practical value, especially in situations where conventional interventions are unavailable or unfeasible. Future research should prioritize well-designed RCTs that address these gaps by incorporating rigorous blinding protocols and validated objective measures. Such improvements will yield stronger evidence to inform clinical decision-making and enhance the credibility of DHTs as interventions for mental health.

The weighted average attrition rate immediately following the intervention was 16.8% for the intervention group. According to a systematic review of physical activity interventions that did not use DHT in nonclinical adults, the attrition rate in the intervention group immediately after the intervention was 25%.^38^ Although all the interventions in this review had durations of 6 months or longer, most of the studies analyzed in our study lasted less than 6 months, making a direct comparison of the results challenging. For physical activity interventions delivered through DHT not limited to physical activity, the attrition rate was 31.7% for online interventions targeting occupational mental health^10^ and 24.1% for smartphone interventions targeting mental health problems.^11^ The lower attrition rate observed in this study compared with that reported in previous research may be attributed to the interventions examined in the present study, which likely involved a higher degree of human interaction, such as laughter yoga sessions^31^ or educational workshops.^33^ Previous reviews have shown that face-to-face interventions have lower attrition rates compared with online interventions,^10^ suggesting that the face-to-face component may have contributed to the lower attrition rate observed in this study.

Only 2 studies^9^^,^^28^ implemented mechanisms to promote adherence. In the online yoga intervention, all participants in the intervention group attended the minimum recommended number of sessions, which may have effectively improved adherence among remote workers.^28^ Social support^39^ and group dynamics^40^ have also been suggested to be effective in promoting adherence to physical activity, and they may have played a role in adherence in this study. However, the generalizability of these results is limited with such sparse data. Given the scant reports on user engagement in digital mental health interventions, it is recommended to report adherence criteria, rate of uptake, level-of-use metrics, duration-of-use metrics, and the number of those who completed intervention.^41^ Accumulating such reports may clarify the factors that affect adherence.

There are several limitations to this study. First, the small number of included studies prevented us from conducting separate analyses based on intervention content or follow-up period, leaving the effects of different programs and their long-term effects unclear. Similarly, variations in participant characteristics and DHT implementation could not be examined due to limited and inconsistent reporting across studies. Second, the meta-analysis for each outcome did not use the same measurement instruments across studies. The use of different scales may have affected the results, owing to the inherent differences in each scale’s characteristics. Third, the assessment of physical activity and sedentary behavior varied considerably across studies. Whereas some studies quantitatively measured these outcomes, others did not assess them at all or relied solely on participation records or qualitative descriptions. Furthermore, as improvements in physical activity outcomes were assessed only in a subset of physical activity interventions delivered through DHT, it remains unclear whether the absence of improvement in mental health outcomes was due to the intervention’s failure to enhance physical activity, or whether physical activity did improve but did not translate into mental health benefits. Fourth, only a few studies included in this review employed interventions incorporating technological algorithms, such as apps, communication robots, and wearable devices. Consequently, the findings primarily reflect the impact of physical activity interventions that do not rely on such algorithms, including electronic messaging and online meetings. Whereas some interventions used DHT as a stand-alone approach (eg, Irvine et al^32^), most employed it to support or deliver physical activity programs. Thus, the observed effects likely reflect both the physical activity component and the supportive role of DHT. Fifth, this review cannot determine whether interventions using DHT are more effective than face-to-face interventions based on the included studies. If physical activity interventions delivered through DHT are more effective or at least as effective as face-to-face interventions, they could serve as evidence to support the broader implementation of DHT-based approaches. However, none of the 8 studies reviewed conducted a direct comparison between face-to-face interventions and those using DHT. Sixth, this review included only RCTs for the systematic review and meta-analysis. However, RCTs do not always reflect real-world conditions. In the future, it may be necessary to consider the body of evidence, including that derived from nonrandomized study designs. Nevertheless, we adopted a meta-analytic approach to quantitatively synthesize the limited available evidence and estimate effect sizes. Seventh, by limiting the studies to those published in English or Japanese, we excluded studies published in other languages, which may have narrowed the scope of the review. Future research should address these limitations and build upon them to enhance the evidence base.

## 5. Conclusion

This systematic review and meta-analysis examined the primary preventive effects of physical activity interventions delivered through DHT on workers’ mental health symptoms. The findings suggest that such combined interventions may have a moderate effect on depression and negative affect, and a small to moderate effect on perceived stress, both observed immediately after the intervention. However, due to the risk of bias and heterogeneity, the overall certainty of the evidence was low, limiting the strength of these findings. In addition, its long-term effects and effects on other mental health symptoms remain unclear. The attrition rate at the end of the intervention was relatively low at 16.8%. Notably, only 2 of the 8 studies provided information on adherence. These findings provide preliminary insights but should be interpreted cautiously due to the small evidence base and variability in study characteristics.

In addition to the main findings, this review also identified several methodological challenges that should be addressed in future research. Future intervention studies should investigate the long-term effects of interventions, examine a broader range of mental health outcomes, and identify factors influencing adherence to interventions delivered through DHT.

## Supplementary Material

Web_Material_uiaf035

## Data Availability

Data are available upon reasonable request.
