# Supplementary material for: Physical activity interventions delivered through digital health technology for improving workers’ mental health symptoms: a systematic review and meta-analysis
Source: J Occup Health. 2025 Jun 30;67(1):uiaf035. doi: 10.1093/joccuh/uiaf035 (PMC12305426; doi:10.1093/joccuh/uiaf035)
Supplement: Web_Material_uiaf035 [file web_material_uiaf035.zip › Appendix 4 Funnel plots.docx]

**Appendix 4.** Funnel Plots


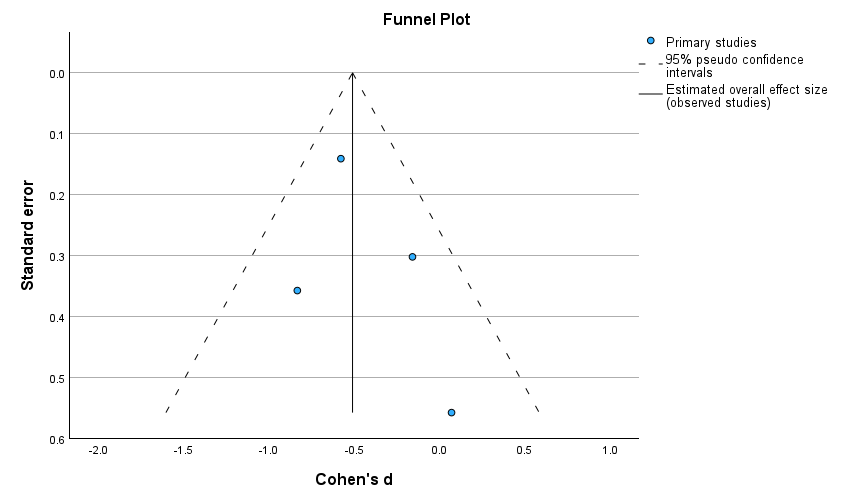


Figure A1. Funnel Plot – depression and negative affect


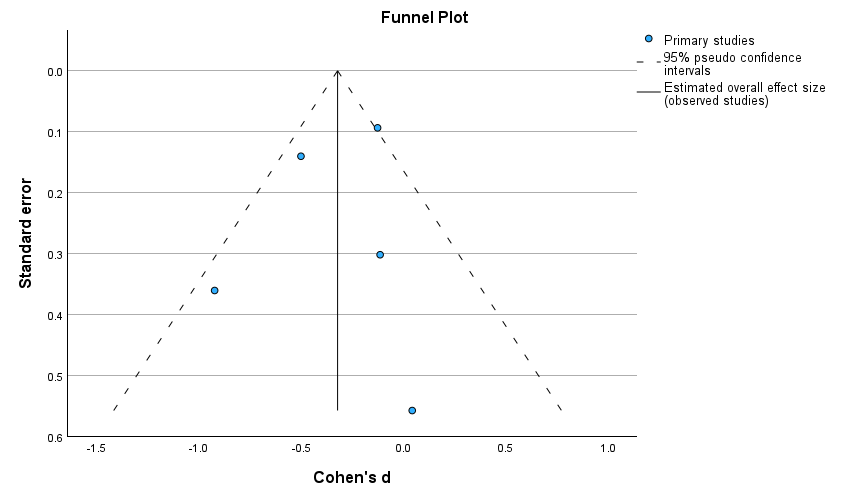


Figure A2. Funnel Plot – perceived stress
