# Supplementary figures and images for: Physical activity interventions delivered through digital health technology for improving workers’ mental health symptoms: a systematic review and meta-analysis
Source: J Occup Health. 2025 Jun 30;67(1):uiaf035. doi: 10.1093/joccuh/uiaf035 (PMC12305426; doi:10.1093/joccuh/uiaf035)

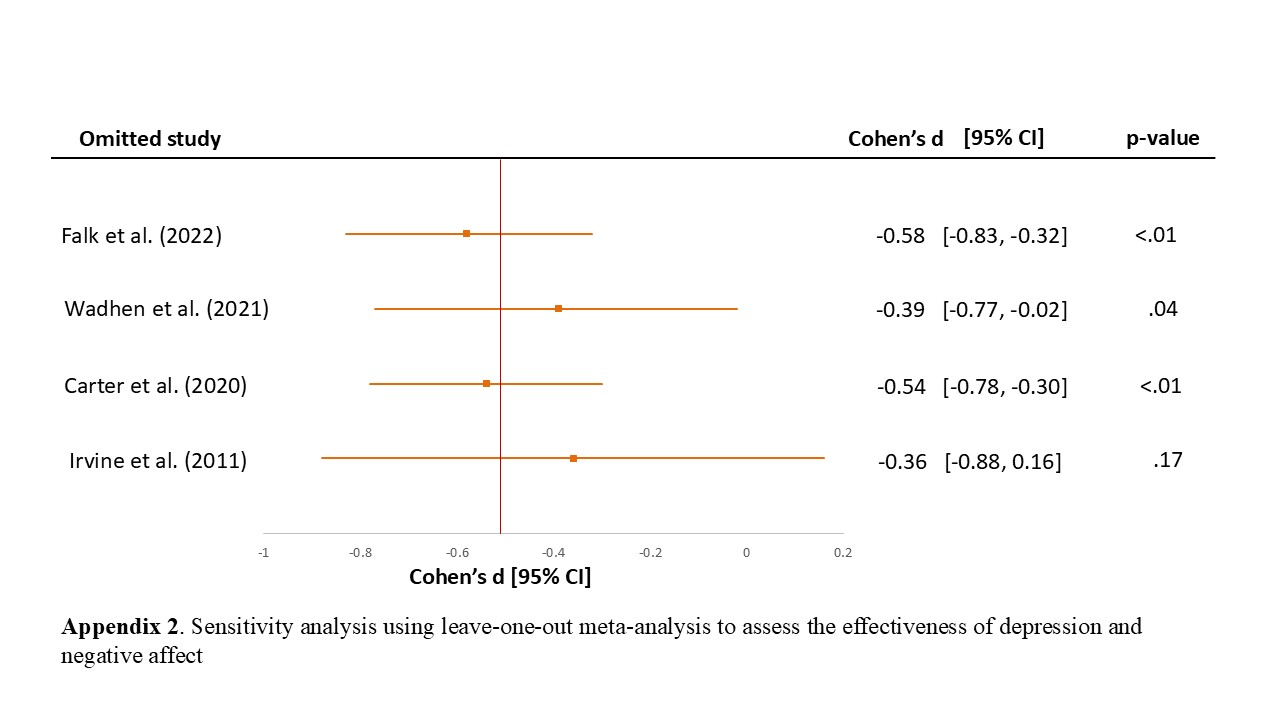

Supplement: Web_Material_uiaf035 [file web_material_uiaf035.zip › Appendix 2.JPG]

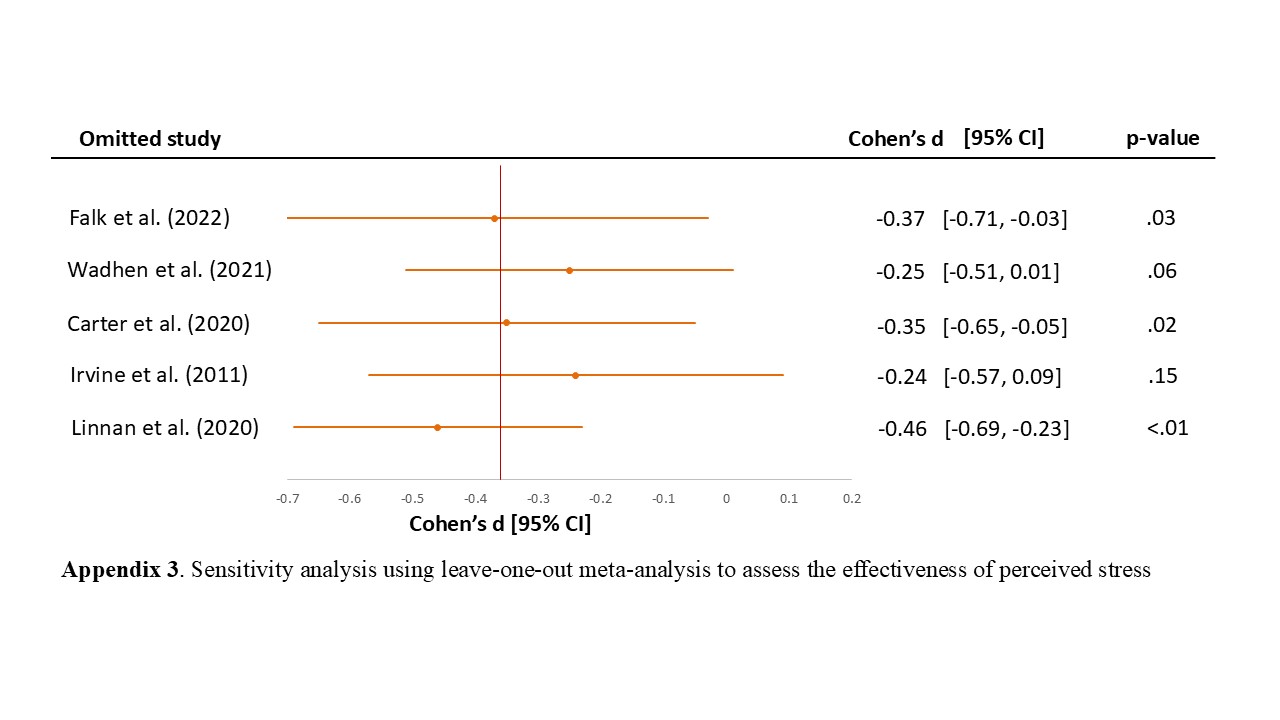

Supplement: Web_Material_uiaf035 [file web_material_uiaf035.zip › Appendix 3.JPG]
